# Systems Biology Approaches for Understanding Metabolic Differences Using ‘Multi-Omics’ Profiling of Metabolites in Mice Fed with Honey and Mixed Sugars

**DOI:** 10.3390/nu14163445

**Published:** 2022-08-22

**Authors:** Xing Zheng, Yazhou Zhao, Nenad Naumovski, Wen Zhao, Guan Yang, Xiaofeng Xue, Liming Wu, Daniel Granato, Wenjun Peng, Kai Wang

**Affiliations:** 1 Institute of Apicultural Research, Chinese Academy of Agricultural Sciences, Beijing 100093, China; 2 University of Canberra Health Research Institute (UCHRI), University of Canberra, Locked Bag 1, Bruce, Canberra, ACT 2601, Australia; 3 Department of Infectious Diseases and Public Health, City University of Hong Kong, Kowloon, Hong Kong 999077, China; 4 Department of Biological Sciences, School of Natural Sciences, Faculty of Science and Engineering, University of Limerick, V94 T9PX Limerick, Ireland

**Keywords:** diet, honey, sugar, gut microbiota, metabolomics, systems biology

## Abstract

Honey is proposed to be the oldest natural sweetener and it is a standard component of several dietary patterns. Recent evidence suggests that replacing sugars, such as fructose, with honey has potential health benefits. In this study, we determined the effects of honey supplementation in mice on cardiometabolic and inflammatory markers and changes in gut microbiota and metabolomic profiles. We compared mice fed a honey diet (1 or 2 g/kg) with those fed an analog diet (mixed fructose, glucose, and sucrose (FSG) solutions) at exact dosages for one month. We found the same blood glucose fluctuating trends for honey- and FGS-fed mice. The honey diets resulted in less weight gain and fewer ballooned hepatocytes. Additionally, honey diets decreased the total serum cholesterol and TNF-α and increased the antioxidant enzyme activity. Each diet type was associated with distinct gut microbiota and metabolomics profiles. Systems biology analysis revealed that *Lactococcus* spp., *Lachnospiraceae* spp., and oleamide had the strongest correlations with lipid metabolic networks. Although in an animal model, this study provides a good understanding of the potential benefits of choosing honey rather than mixed sugars in regular dietary patterns.

## 1. Introduction

Sweet foods have always been a part of the human diet. An enjoyment of sweet tastes is present at birth, and a preference for sweet tastes over bland tastes (water) is common during the developmental period. Honey is one of the longest-used natural sweeteners, and its use dates back to prehistoric times. However, the success of extracting and refining sugars from sugarcane and beets on large industrial scales has greatly influenced the production of sweet foods, making sweets inexpensive and partly replacing the importance of honey [1]. In the 19th and 20th centuries, the large-scale development of sugar syrup resulted in less expensive production of common sweeteners. Since relatively recently, an increasing number of sugar substitutes have been developed that can successfully mimic the perception of sweet taste (sucralose or sugar alcohol), in addition to providing less energy than honey or sucrose. Although non-nutritive sweeteners are still considered to be safe and well-tolerated, the findings of relatively recent studies remain controversial, indicating that their intake may be associated with a higher risk of hypertension and cardiovascular events [2].

Honey is a naturally produced food product resulting from the combination of plant nectar, and bees′ secretions are deposited in the honeycomb for maturation. Honey is also regarded as one of the oldest food products used for the management of several health conditions in addition to being a regular component of the diet. Due to its notable antioxidant, anti-microbial, and anti-inflammatory effects, honey has become an attractive functional food to decrease the harmful effects of oxidative stress and inflammation [3]. Relatively recent findings from extensive prospective cohort studies have indicated that the consumption of honey might not only reduce the risk of different metabolic-related disorders but also improve glycemic control and nonalcoholic fatty liver disease, and display lipid-lowering effects [4].

Human clinical trials have shown that the sucrose diet leads to a significant increase in the energy, carbohydrate, and fat intakes compared to the honey diet. Furthermore, the total serum cholesterol, triglycerides, and low-density lipoproteins were increased, and high-density lipoproteins were decreased, in sucrose diet subjects [5]. In addition, the daily consumption of fructose-containing beverages caused disturbances in hepatic lipid metabolism and increased the basal lipogenic capacity. However, the honey diet did not cause elevated serum TGs in rats when it was used as a source of fructose [6]. Compared to the starch-based diet, fructose induced lower plasma alpha-tocopherol levels in rats, and there was less protection against lipid peroxidation [7]. A high-fructose diet elevated fasting glucose in rats, and rats on a high-fructose diet had increased expression levels of genes encoding inflammatory factors. Therefore, we hypothesized that replacing fructose with honey in the diet has potential nutritional benefits.

We conducted a systematic study of gut microbial and serum metabolic indicators and the differences in blood glucose, body weight, inflammatory response, antioxidant activity, and serum lipid content associated with honey and mixed-sugar-solution diets. We aimed to highlight the differences between natural mature honey and sugar solutions based on metabolomics and gut microbiota sequencing technology, and analyze the murine model′s systemic regulatory mechanisms.

## 2. Materials and Methods

### 2.1. Chemical Reagents

We ordered formic acid and acetonitrile from Fisher Scientific Inc. (Pittsburgh, PA, USA). We bought fructose, glucose, and sucrose from Shanghai Macklin Biochemical Co., Ltd. (Shanghai, China). Entirely capped chaste (*Vitex agnus-castus* L.) monofloral honey samples were obtained from *Apis mellifera* L. colonies in the Miyun District, Beijing, China (latitude, 117.044996°; longitude, 40.440968°) at the end of July 2020. Honey samples were stored at −20 °C until use.

### 2.2. Preparation of Honey and Honey Analog (Mixed-Sugar) Diets

Mature chaste honey was assessed by a Nexera UHPLC LC-30A (equipped with RID-10A, Shimadzu, Japan) and contained 39% fructose, 28% glucose, and 4% sucrose [8]. The low-dose and high-dose honey diet groups were given a gavage of 0.1 or 0.2 g/mL (pure water as solvent) honey solution, respectively. In addition, low doses of mixed sugar (0.039 g/mL fructose, 0.028 g/mL glucose, 0.004 g/mL sucrose) and high doses of mixed sugar (0.078 g/mL fructose, 0.056 g/mL glucose, 0.008 g/mL sucrose) dissolved in pure water were utilized to create the mixed-sugar diets.

### 2.3. Animal Experiment

**Figure 1:**
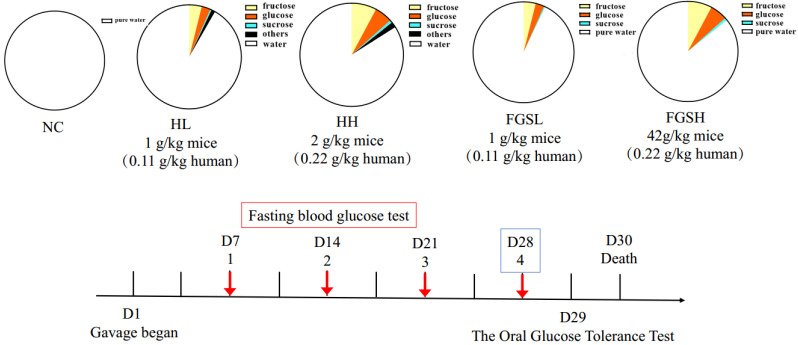
Timeline of events and experimental design used.

We ordered 4~6-week-old male mice (ICR strains) from Beijing Vital River Laboratory Animal Technology Co., Ltd. (Beijing, China). Mice were kept in specific pathogen-free conditions. The experimental protocol was approved by the Animal Ethics Committee (AEC NO:20210115) of the Institute of Apicultural Research of the Chinese Academy of Agricultural Sciences. The animal experiments were performed in the Department of Experimental Animals, Zhejiang Academy of Traditional Chinese Medicine (Hangzhou, Zhejiang, China, AEC code: 2022035). The dosages were chosen based on our previous studies [9]. A total of 40 mice were distributed into five groups (n = 8 animals) and were fed once a day by intragastric gavage: (A) normal diet group (ND): mice were fed a standard laboratory chow diet and water; (B) low-dose honey diet group (HL): mice were fed a low-dose honey diet (1 g/kg); (C) high-dose honey diet group (HH): mice were fed a high-dose honey diet (2 g/kg); (D) mixed-sugar low-dose diet (mixed sugar with fructose, glucose, and sucrose low-dose, FGSL): mice were fed a low-dose sugar diet (39% fructose, 28% glucose and 4% sucrose, 1 g/kg); (E) mixed-sugar high-dose diet (mixed sugar with fructose, glucose and sucrose high-dose, FGSH): mice were fed a high-dose sugar diet (39% fructose, 28% glucose and 4% sucrose, 2 g/kg mice) (Table 1 and Figure 1). The mice started fasting every Thursday night, and they were weighed and given treatment at 8:30 every Friday. The fasting blood glucose was measured at 15:30. An oral glucose tolerance test (OGTT) was conducted after 4 weeks of treatment. On the last day, the body weight was measured when the mice had an empty stomach, and serum, organ, tissue, and fecal samples were collected from each mouse for subsequent analyses. The formula to calculate the visceral index = organ weight/body weight × 100%.

### 2.4. Oral Glucose Tolerance Test

The OGTT was assessed 29 days after the honey/sugar diet intervention [10]. The mice fasted for 8 h and then consumed 2.5 g/kg body weight of D-glucose by orally gavage, followed by a collection of blood samples from the tail vein at different time points. A glucometer (Accu-Chek Performa, Roche Diagnostics GmbH, Mannheim, Germany) was used to measure the glucose levels. The area under the curve (AUC) for the OGTT was also calculated.

### 2.5. Histological Analysis

The liver and abdominal adipose tissues were fixed in 4% paraformaldehyde solution [11]. The paraffin sections (3 μm) were stained with hematoxylin and eosin (H&E) solution (Sigma-Aldrich, St. Louis, MO, USA) and observed under an optical microscope (Nikon Eclipse E100, Tokyo, Japan). The size and number of adipocytes and hepatocytes were analyzed using ImageJ software 1.48 (Biteplane AG, Switzerland).

### 2.6. Analysis of Lipids, Inflammation, and Antioxidant Factors in Serum

The concentrations of total serum cholesterol (T-CHO), triglycerides (TGs), high-density lipoprotein cholesterol (HDL-C), low-density lipoprotein cholesterol (LDL-C), and the activity of superoxide dismutase (SOD) and glutathione (GSH) were measured using commercially available kits (Nanjing Jiancheng Bioengineering Institute, Nanjing, China). Serum tumor necrosis factor (TNF-α) was examined with commercial ELISA kits (CUSABIO Life Sciences, Wuhan, China). The concentration of serum plasma lipopolysaccharide (LPS) was measured based on an End-Point Chromogenic Endotoxin Test Kit (Xiamen Bioendo Technology Co., Ltd., Xiamen, China).

### 2.7. Gut Microbiota Analysis

For gut microbiota analysis, the DNA of feces was extracted from 100 mg cecal digesta samples using the TIANamp Stool DNA Kit (TIANGEN Biotech Co., Ltd., Beijing, China). Extracted DNA was detected using a NanoDrop 2000 UV–vis spectrophotometer (Thermo Fisher Scientific, San Jose, CA, USA). The extracted DNA was amplified using conventional bacterial primers 338F (5′-ACTCCTACGGGAGGCAGCAG-3′) and 806R (5′-GGACTACHVGGGTWTCTAAT-3′). The DNA fragments were sequenced on an Illumina MiSeq PE300 platform. The RDP classifier (V 2.2) annotated each sequence for species classification [9]. We used the online web analysis database Ultra-Fast Sequence Analysis (http://www.drive5.com/usearch/, accessed on 1 March 2022) to assign ≥97% of the similar sequences of the intestinal flora to the same operational taxonomic units (OTUs). Alpha diversity analysis (Student’s t-test) was performed using the online database Mothur (https://www.mothur.org/wiki/Download_mothur, accessed on 2 March 2022) to measure the intra-individual community diversity. The greater the Ace, Chao, and Shannon indexes, the greater the community diversity of an individual. Moreover, the greater the Simpson index, the lower the community diversity. The beta diversity analysis showed the variability of the flora between the groups. For the beta-diversity analysis, we used the online database Quantitative Insights Into Microbial Ecology (http://qiime.org/install/index.html, accessed on 3 March 2022) to analyze the difference in the genus-level projections to latent structures-discrimination analysis (PLS-DA) and phylum-level community abundance. The differences between treatment groups at the genus level were analyzed using one-way ANOVA and Wilcoxon’s rank-sum test [12].

### 2.8. Metabolite Profiling

For the exact extraction method, we referred to Guo et al. [8]. In brief, serum samples (100 μL) were extracted with 400 µL of methanol and acetonitrile (1:1, v/v), and the extraction efficiency was facilitated by vortexing and sonication. The samples were centrifuged, and the supernatant was dried. The serum was then re-solubilized with methanol and acetonitrile (1:1, v/v). Finally, the supernatant extract was filtered (0.22 μm nylon membrane) before metabolite analysis. The instrumentation was based on an Agilent HPLC system combined with quadrupole time-of-flight mass spectrometry (HPLC-Q-TOF/MS, 6545, Agilent Technologies, Palo Alto, CA, USA). The test method was also adopted in our previous study [9].

### 2.9. Statistical Analyses

Data were assessed using SPSS17.0 (Chicago, IL, USA). One-way ANOVA and Duncan′s post-hoc test were used to determine differences between groups, expressed as the mean ± standard deviation (SD), with *p* values < 0.05 considered significant.

## 3. Results

### 3.1. Effects of Honey and Mixed-Sugar Exposure on Blood Glucose, Body Weight, Adipocytes, Serum Lipids, Inflammatory Response, and Antioxidant Activity

**Figure 2:**
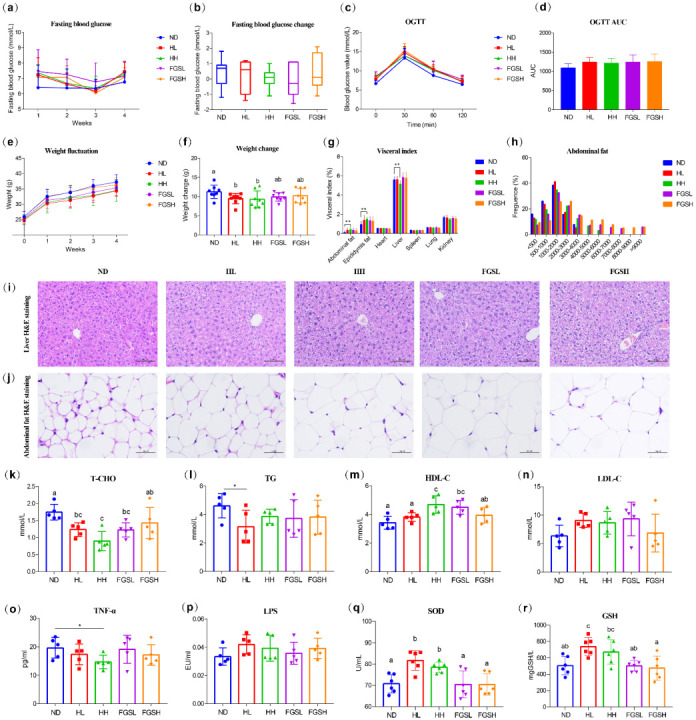
Effects of honey and mixed-sugar feeding on glucose homeostasis, body weight, liver and abdominal fat accumulation, visceral index, serum lipid and inflammatory factors, and oxidative stress levels. Fasting blood glucose levels (**a**) and their change values (**b**) were measured weekly over four weeks. Results of the OGTT (**c**) and its AUC (**d**) were detected after a long gavage period. Body weight fluctuation (**e**) and change (**f**) were measured weekly for four weeks. Visceral index (**g**) was detected after a long feeding period. Liver (**i**) and abdominal fat (**j**) cell morphology were assessed using hematoxylin and eosin staining. Abdominal adipocyte size (**h**) was evaluated by ImageJ software. Scale bar, 50 μm. Lipid concentrations are presented of T-CHO (**k**), TGs (**l**), HDL-C (**m**), and LDL-C (**n**). Cytokine concentrations are presented of TNF-α (**o**) and LPS (**p**). Serum oxidative stress levels were analyzed in terms of the enzyme activity—SOD (**q**) and GSH (**r**). Data are expressed as the mean ± SD. Graph bars (**f**,**k**,**m**,**q**,**r**) with different letters on top correspond to statistically significant results (*p* < 0.05) based on a one-way ANOVA. In contrast, bars with the same letter correspond to results that showed no statistically significant difference. Differences in the graph bars (**g**,**l**,**o**) were analyzed using one-way ANOVA (* *p* < 0.05, ** *p* < 0.01).

**Figure A1:**
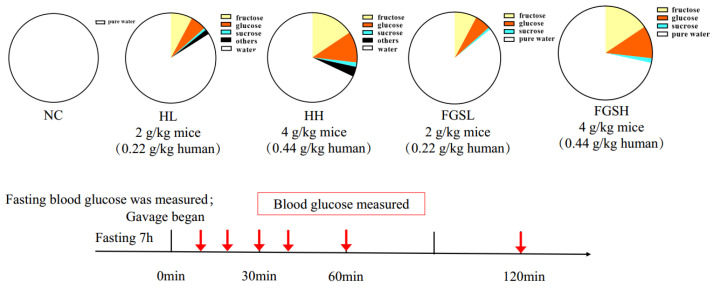
Single-dose timeline of events and experimental design used.

**Figure A2:**
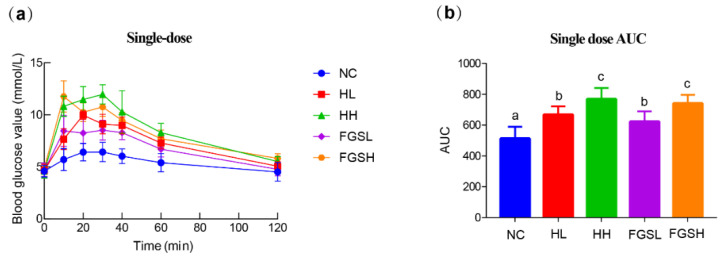
Honey and sugar feeding to mice affected glucose homeostasis over 2 h. Blood glucose value changes (**a**) and the AUC (**b**) were monitored in the single dose test. Data are expressed as the mean ± SD. Graph bars with different letters on top correspond to statistically significant results (*p* < 0.05) based on a one-way ANOVA. In contrast, bars with the same letter correspond to results that showed no statistically significant differences.

We administered a single dose before the long-term feeding experiment (Table A1 and Figure A1) and observed that HH and FGSH were associated with higher blood glucose levels over 120 min, as shown in the line chart (Figure A2a) and the area under the curve (AUC) (Figure A2b). In the four-week honey/mixed-sugar feeding trial, the fasting blood glucose concentrations were higher in the honey and mixed-sugar diets than in the NC (Figure 2a). Still, there was no significant difference between the groups (*p* = 0.271) (Figure 2b). The fasting blood glucose concentrations fluctuated within the normal range in each group of mice. Therefore, honey and mixed-sugar feeding in this experiment did not affect the body′s ability to regulate the blood glucose concentration.

Compared to the other groups, the honey diet was associated with the lowest weight gain (Figure 2e), and the weight change within four weeks was notably lower than that of the NC (*p* = 0.036) (Figure 2f). Additionally, the visceral index indicated that the abdominal (*p* = 0.020) and epididymal fat (*p* = 0.040) indexes were considerably lower in NC than in HL. The liver index was observably lower in HL than in NC (*p* = 0.011). Indices of other organs, such as the heart, spleen, lung, and kidney, did not differ markedly (Figure 2g). The results of our experiment showed that the mixed-sugar diet contained a more significant number of ballooned hepatocytes in the liver compared to the control and honey diets (Figure 2i). There was a consistent trend in the abdominal adipocyte cross-sectional area ratio between the low-dose honey diet group and the control group (Figure 2j,h). However, the dietary sugar groups had larger abdominal fat cells than the honey feeding and control groups (Figure 2j,h).

The levels of T-CHO (Figure 2k) and TG (Figure 2l) were significantly reduced in the serum of mice fed HL compared to those in the NC group (*p* = 0.006). The HH and FGSL diets significantly increased HDL-C (Figure 2m) compared to the effects of the control diet (*p* = 0.006). Nevertheless, there were no considerable differences in T-CHO, TGs, HDL-C, or LDL-C between the FGSH and NC diets (Figure 2k–n). Additionally, no groups had considerable differences in serum LDL-C concentrations (Figure 2n). In all experimental groups, inflammatory signaling during gavage was assessed by quantifying the serum levels of TNF-α and LPS. We observed that the HH mice showed a considerable decrease in serum TNF-α compared to NC (Figure 2o) (*p* = 0.033). However, each group′s serum LPS levels remained the same (Figure 2p). In addition, HL and HH treatment significantly increased the concentrations of SOD and GSH compared to the effects in the NC group (*p* < 0.05) (Figure 2q,r).

### 3.2. Honey and Mixed Sugar Were Associated with Distinct Gut Microbiota Compositions

**Figure 3:**
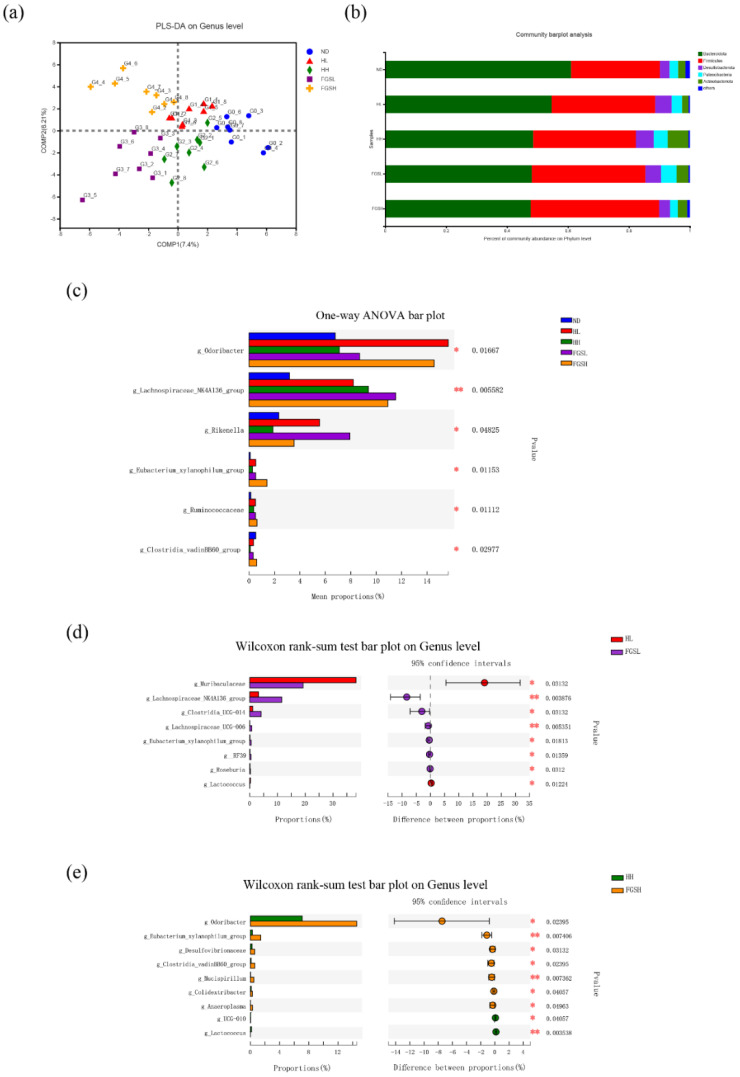
Honey and mixed-sugar feeding resulted in distinct microbiota compositions. Partial least squares-discrimination analysis (PLS-DA) plots based on unweighted UniFrac (**a**). Bacterial community bar plot analysis at the phylum level of gut bacteria from different groups of mice (**b**). A one-way ANOVA bar plot was used to analyze the differences between species groups at the genus level (**c**). Significant differences in intestinal flora species at the genus level were identified between low-dose honey and low-dose sugar diet groups and high-dose honey and high-dose sugar diet groups by Wilcoxon’s rank-sum test bar plot (**d**,**e**). Significance analysis involved, * *p* < 0.05, ** *p* < 0.01.

**Figure A3:**
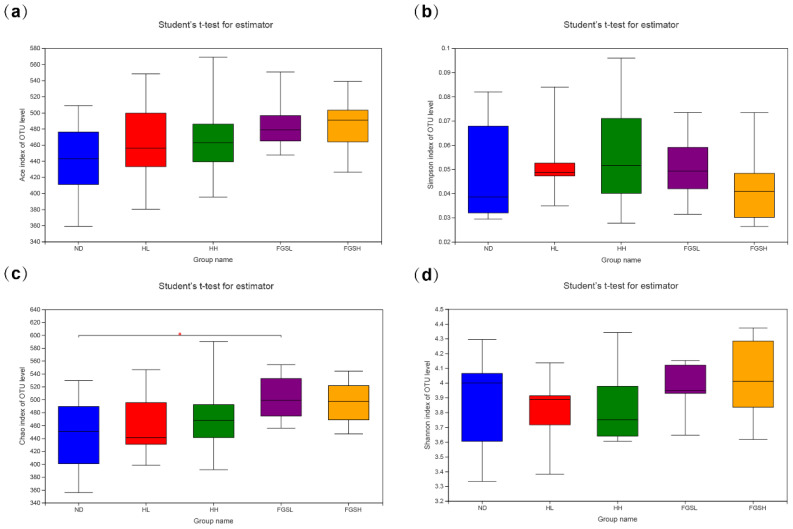
Alpha diversity analysis of intestinal flora in mice. Alpha diversity of Ace (**a**), Simpson (**b**), Chao (**c**), and Shannon indexes (**d**) at the OTU level. Differences in the graph bars were analyzed using one-way ANOVA (* *p* < 0.05).

The alpha diversity at the OTU level indicated that the gut microbiota composition of each group was similar (Figure A3a,b,d). The Chao index of the FGSL was higher than that of the NC group; therefore, the intestinal flora composition of FGSL feeding mice was more complex (Figure A3c). Through PLS-DA mapping (Figure 3a), it was possible to observe that the honey and mixed-sugar diets produced a significantly fragmented distribution of intestinal flora at the genus level. At the phylum level, *Bacteroidetes* (61.03%) and *Firmicutes* (29.11%) were significant in the intestinal bacteria of the NC group, followed by *Desulfobacteria* (3.2%), *Patescibacteriota* (2.8%), and *Actinobacteria* (2.4%) (Figure 3b). Moreover, a one-way ANOVA at the genus level showed that the honey and mixed-sugar diets caused considerable changes in six intestinal bacteria. Visible increases in *Odoribacter* spp., *Lachnospiraceae_NK4A136* group, *Rikenella*, *Eubacterium_xylanophilum* group, and *Ruminococcaceae* spp. were found in the honey- and mixed-sugar-fed mice compared to the NC group (*p* < 0.05) (Figure 3c). However, the HL group showed a noticeable increase in *Muribaculaceae* and a decrease in the *Lachnospiraceae NK4A136* group, *Clostridia UCG-014,* and *Lachnospiraceae UCG-006* compared to the FGSL groups (*p* < 0.05) (Figure 3d). The HH treatment provoked a noticeable decrease in *Odoribacter*, *Eubacterium xylanophilum* group, *Desulfovibrionaceae*, *Clostridia vadinBB60* group, and *Mucispirillum* compared to the FGSH groups (*p* < 0.05) (Figure 3e).

### 3.3. Mice Fed Honey and Mixed-Sugar Solutions Had Different Metabolite Profiles

**Figure 4:**
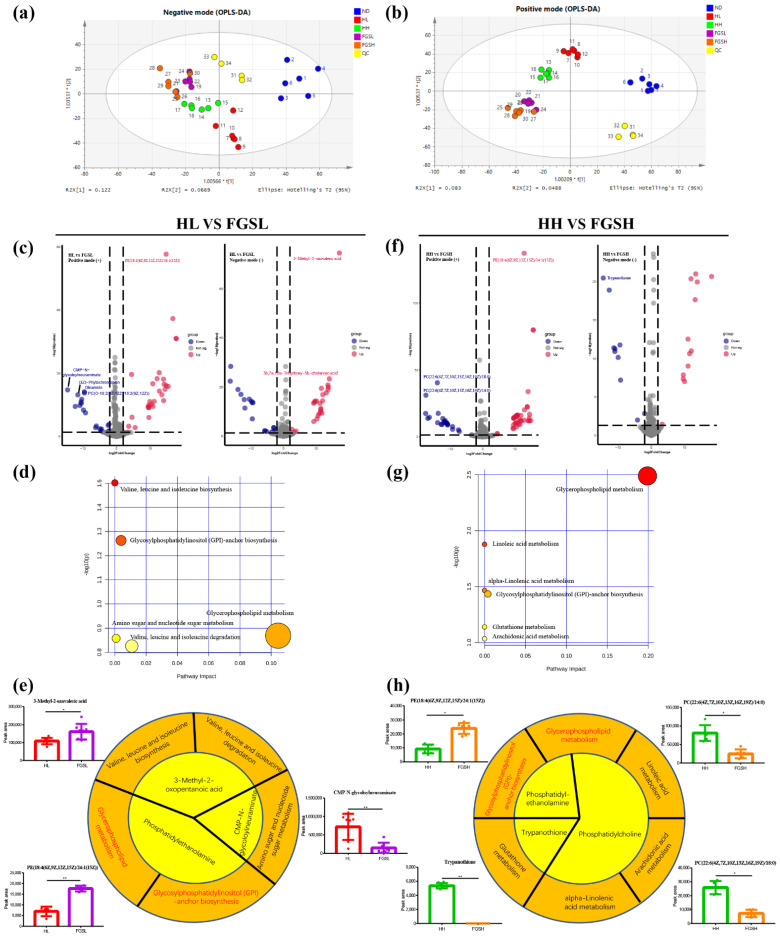
Honey and sugar diets have different metabolite profiles. Orthogonal projections to latent structures-discrimination analysis (OPLS-DA) score plots of metabolomics data in negative and positive modes, respectively (**a**,**b**). Volcano plot screening showed that the honey and sugar diet mice serum had different metabolite biomarkers (**c**,**f**). Moreover, the bubble chart indicates that they perturbed metabolic pathways (**d**,**g**). The biomarkers involved in different metabolic pathways were diverse in HL and FGSL (**e**,**h**). Graph bar (e and h) differences were analyzed using one-way ANOVA (* *p* < 0.05, ** *p* < 0.01). The text continues here in the Figure 2 and Table 1 captions.

Orthogonal projections to latent structures-discrimination analysis (OPLS-DA) score plots of metabolomics data and pathway analysis in honey- and mixed-sugar-fed mice are shown in Figure 4a,b. The NC, HL, HH, FGSL, and FGSH diets could be readily separated using the first two components of OPLS-DA, suggesting that these diet treatments induced conspicuous perturbation of intracellular metabolites.

Positive-ion-mode volcano plot screening showed significantly downregulated CMP-N-glycoloylneuraminate, (3Z)-phytochromobilin, PC(O-18:2(9Z,12Z)/18:2(9Z,12Z)), and oleamide and observably upregulated PE (18:4(6Z,9Z,12Z,15Z)/24:1(15Z)) in FGSL-fed mice compared to mice in the HL group. Negative-ion-mode volcano plot screening revealed markedly upregulated 3-methyl-2-oxopentanoic acid and 3b,7a,12a-trihydroxy-5b-cholanoic acid in FGSL-fed mice compared to HL-fed mice (Figure 4c,e). Based on the KEGG pathway database, the metabolites notably changed between the HL and FGSL groups were highly associated with glycerophospholipid metabolism; glycosylphosphatidylinositol (GPI)-anchor biosynthesis; valine, leucine, and isoleucine biosynthesis; valine, leucine, and isoleucine degradation, and amino sugar and nucleotide sugar metabolism in mouse serum (Figure 4d,e).

Moreover, the positive-ion-mode volcano plot screening showed significantly downregulated PC (22:6(4Z,7Z,10Z,13Z,16Z,19Z)/18:0) and PC (22:6(4Z,7Z,10Z,13Z,16Z,19Z)/14:0) and observably upregulated PE (18:4(6Z,9Z,12Z,15Z)/24:1(15Z)) in FGSH-fed mice compared to HH-fed mice. Further to this, negative-ion-mode volcano plot screening revealed markedly downregulated trypanothione in FGSH-fed mice compared to HH-fed mice (Figure 4f,h). According to the KEGG database, the metabolites significantly changed between the HH and FGSH interventions were highly enriched in glycerophospholipid metabolism and glycosylphosphatidylinositol-anchor biosynthesis as linoleic acid metabolism pathways (Figure 4g,h).

### 3.4. Systems Biology Approaches to Interpreting the Effects of Honey and Mixed-Sugar Diets on the Organism

**Figure 5:**
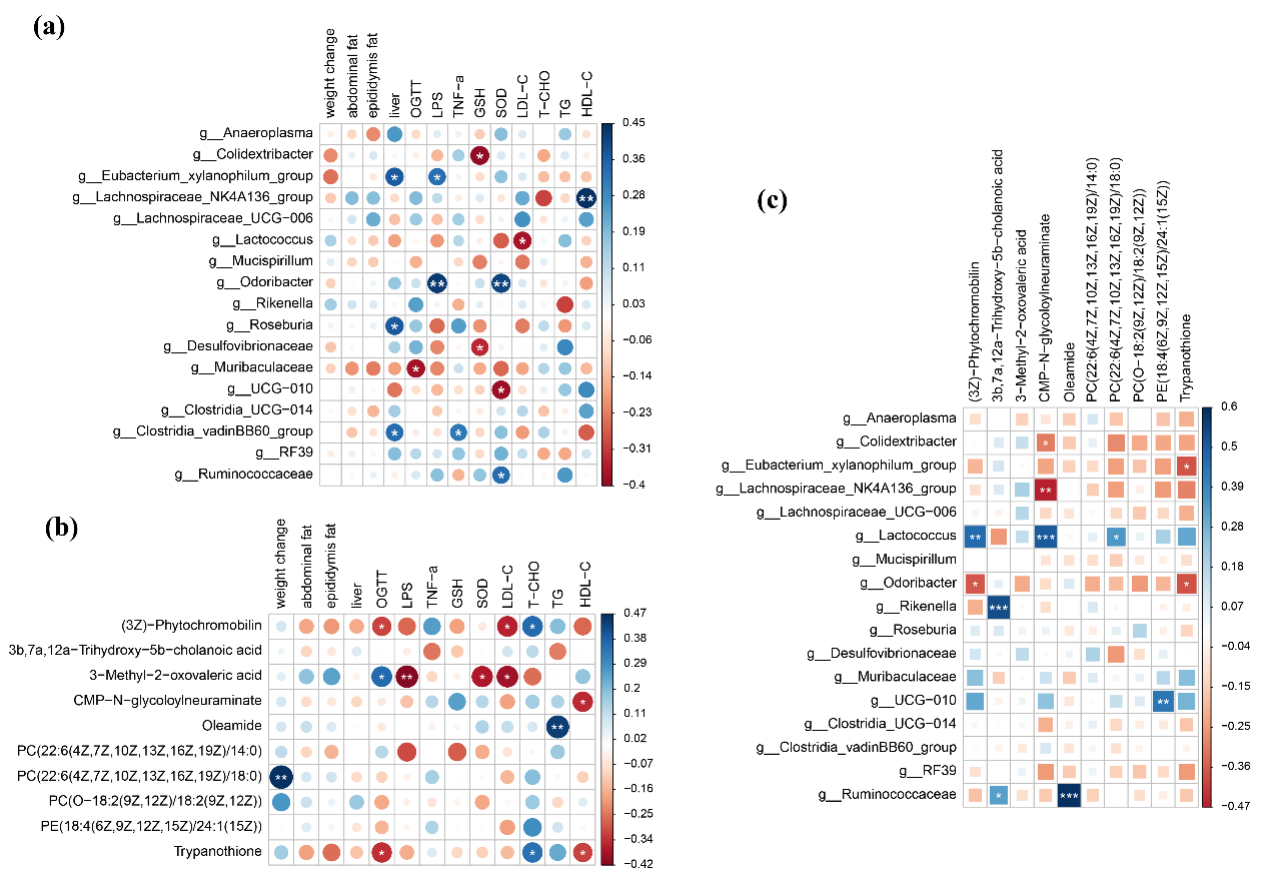
Association of clinical phenotypes with gut microbiota and serum metabolites from a systems biology perspective. Correlation heatmap showing the functional relationship between the clinical index and disturbed gut bacterial genera (**a**). Correlation heatmap showing the functional relationship between the clinical index and altered serum metabolites (**b**). Correlation heatmap between the altered gut microbiome and metabolites (**c**). Blue represents positive associations. Red denotes inverse associations. Heatmap differences were analyzed using an R package (* *p* < 0.05, ** *p* < 0.01).

Finally, based on correlation analysis approaches, 17 different bacteria were obtained by performing one-way ANOVA and Wilcoxon’s rank-sum test analysis on the genus-level intestinal bacteria in each group. Spearman’s correlation analysis was performed with the detection indicators (Figure 5a). After one month of consuming honey and sugar diets, the weight change, abdominal fat, epididymal fat, and T-CHO and TG concentrations were not associated with intestinal bacteria in any group of mice. *Eubacterium xylanophilum* group, *Roseburia,* and *Clostridia vadin BB60* group in the gut were positively related to the liver weight in mice. The OGTT showed a markedly negative correlation with *Muribaculaceae*. The inflammatory factor LPS was positively correlated with the *Eubacterium xylanophilum* group and *Odoribacter*. Moreover, TNF-α was positively associated with the *Clostridia_vadinBB60_*group. The antioxidant GSH was inversely related to *Colidextribacter* and *Desulfovibrionaceae*. The antioxidant enzyme SOD was positively correlated with *Odoribacter* and *Ruminococcaceae* and negatively correlated with *UCG-010*. The lipid metabolism factor LDL-C appeared to be significantly inversely correlated with *Lactococcus* intestinal bacteria. *Lactococcus* spp. and *Lachnospiraceae* spp. had the greatest correlations with lipid metabolic networks. Simultaneously, HDL-C demonstrated a notably positive relationship with the intestinal bacteria *Lachnospiraceae_NK4A136* group. A volcano plot screened out the top ten main serum metabolites with differences in the control and treatment groups. Spearman’s correlation analysis with the clinical indicators showed that abdominal fat, epididymal fat, liver weight, TNF-α, and GSH were not significantly related to any metabolites (Figure 5b). Weight change was positively related to PC (22:6(4Z,7Z,10Z,13Z,16Z,19Z)/18:0). (3Z)-Phytochromobilin and trypanothione were inversely correlated with the OGTT results, but 3-methyl-2-oxovaleric acid was positively related to the OGTT results. Meanwhile, 3-methyl-2-oxovaleric acid was negatively correlated with LPS and SOD. (3Z)-Phytochromobilin and 3-methyl-2-oxovaleric acid were inversely correlated with LDL-C. (3Z)-Phytochromobilin and trypanothione were negatively correlated with T-CHO. The serum marker oleamide has a significant positive correlation with the lipid factor TG. CMP-N-glycoloylneuraminate and trypanothione showed a markedly negative correlation with HDL-C.

We further correlated the gut microbiota and serum metabolites using Spearman’s correlation analysis (Figure 5c). *Colidextribacter* showed a significantly negative relationship with CMP-N-glycoloylneuraminate. *Eubacterium xylanophilum* group was negatively correlated with *trypanothione*. *Lachnospira_NK4A136* group was negatively correlated with CMP-N-glycoloylneuraminate. *Lactococcus* demonstrated a markedly positive correlation with (3Z)-phytochromobilin, CMP-N-glycoloylneuraminate, and PC (22:6(4Z,7Z,10Z,13Z,16Z,19Z)/18:0). *Odoribacter* appeared to have a notably negative correlation with (3Z)-phytochromobilin and trypanothione. *Rikenella* was significantly positively correlated with 3b,7a,12a-trihydroxy-5b-cholanoic acid. *UCG-010* was observably positively correlated with PE (18:4(6Z,9Z,12Z,15Z)/24:1(15Z)). *Ruminococcaceae* showed a significantly positive correlation with 3b,7a,12a-trihydroxy-5b-cholanoic acid and oleamide.

## 4. Discussion

Sweetness appeals to everyone because eating sugar prompts the brain to produce large amounts of dopamine, the chemical that causes feelings of pleasure and happiness. The most common natural sweeteners used commercially are sucrose, fructose, glucose, and their mixed form (syrup). Yet, their excessive consumption has been found to cause obesity, type II diabetes, dental caries, and cardiometabolic diseases [13]. As a natural product of complex composition, honey contains monosaccharides, disaccharides, and oligosaccharides. In addition, honey contains a number of other phytochemicals such as phenolic acids and flavonoids, minerals, vitamins, proteins, amino acids, enzymes, organic acids, and volatile compounds [14]. Food manufacturers and consumers have not fully embraced honey as a sugar substitute, and few studies have systematically com-pared honey and sugar diets in mice. In this study, based on a systems biology approach, it was observed that a diet of honey and mixed sugar (including 39% fructose, 28% glucose, and 4% sucrose) affected the body weight and fat metabolism in mice, and the two diets resulted in different intestinal tract flora and serum metabolites.

Since mature chaste honey and mixed sugar contain 28% glucose, the honey and sugar diet caused considerably higher blood glucose levels than NC in the acute gavage test. However, normal mice could regulate their blood sugar. After 120 min, the blood sugar values of each group were similar and tended to normalize. Four-week honey and sugar gavage experiments revealed that the two diets did not cause significant changes in fasting blood glucose values or oral glucose tolerance. In this case, the honey polyphenols and other compounds did not suppress the glucose response. This was expected as the solutions were prepared based on the honey composition. Studies among 60 adult patients with impaired fasting glucose showed that honey does not affect the fasting blood glucose or oral glucose tolerance [15]. This is consistent with our findings. However, research in models of diabetes and obesity has reported that dietary honey can reduce fasting blood glucose and HbA1c [16]. Multiple mechanisms explain the ability of honey to control blood sugar. Firstly, fructose and oligosaccharides may slow gastric digestion and decrease intestinal glucose absorption, and specific oligosaccharides act as prebiotics to promote an increase in beneficial gut microbes. In addition, polyphenols and flavonoids in honey have antioxidant and hypoglycemic properties, which may improve the beta-cell function and hepatic insulin sensitivity [17]. This experiment utilized the doses previously reported [9], and to avoid an impact on the later oral glucose tolerance (2.5 g/kg/mouse), we set the highest dose to 2 g/kg/mouse. Due to the low intake of honey and insufficient gavage time in our setting, we did not observe a significant effect of honey on blood sugar levels (all *p*s > 0.05). Indeed, animal studies, especially those studying homeostasis, are moving toward utilizing both sexes to better model diseases. We acknowledge that this experiment was limited to male mice only, and it is not clear whether chaste honey and mixed-sugar diets have similar results for female mice. Further studies are warranted to compare the sex differences to test whether sugar/honey diets still have differences. Under free-feeding conditions, there was no significant variability in food and water intake among the groups of mice that we monitored weekly. However, the honey diet significantly slowed weight gain, which is consistent with other studies [18]. Gelam honey fed to obese rats reduced high-fat diet-induced weight gain [19], which may be related to the absorption of nutrients [20]. Since we neglected to monitor the weight of mouse feces daily, it was not possible to ensure that the ingested honey was completely absorbed. Although the liver weight of the HH-fed mice was significantly lower than that of NC after four weeks, no obvious abnormality was observed in liver H&E staining. Unexpectedly, the abdominal fat and epididymal fat of mice fed 2 g honey/kg mouse were observably higher than those in NC. This contradicts the findings of many previous studies. Romero-Silva posited that honey-fed rats had notably fewer fat cells after two months than sugar-fed rats [21]. It has been hypothesized that consuming honey may facilitate the conversion of excess calories to dissipated energy rather than storage as fat in the body [22]. From the H&E staining of abdominal fat (Figure 2j,h), it could be seen that the 1 g honey/kg diet did not significantly increase the volume of adipocytes. Still, the importance of epididymal adipocytes in the sugar diet was markedly higher than in the NC group, indicating that the sugar diet would promote fat accumulation. This may be because 39% fructose rapidly induces the secretion of intestinal fructose transporters and hepatic lipid-producing enzymes, promoting energy storage [23].

The HL treatment (1 g/kg) significantly reduced serum TGs, possibly due to the potential impact of indigestible oligosaccharides in honey that affected TG concentrations. Moreover, Gelam honey observably minimizes serum TG levels in healthy male Sprague Dawley rats [24]. Bioactive substances in honey, such as antioxidants, can reduce serum TG concentrations. We found that the HH treatment (2 g/kg) markedly decreased serum T-CHO (51.5%) and increased the HDL-C concentration (137%) compared to the control group. This is in contrast to previous findings [18]. Nevertheless, the findings of a relatively recent systematic literature review of 23 existing studies and current controlled trials assessing the effect of the honey diet on blood lipid levels in adults propose that honey does not have a prominent lipid-lowering effect [25]. In addition, there was no considerable difference in the average levels of LDL, HDL, T-CHO, and TGs in healthy rats treated with acacia, astragalus, and artificial honey and the control group at the end of the treatment period [26]. Inflammation is produced by immune cells secreting proinflammatory cytokines. The findings of our study showed that HH treatment (2 g/kg) can markedly reduce the concentration of TNF-α (74.7%) in serum, potentially due to the inhibition of the activity of the TNF-α-converting enzyme. [27]. Disturbance of energy metabolism in serum occurs after exposure to honey and sugar solutions. Sugar intake can induce oxidative damage, and honey consumption may induce antioxidative responses in the liver [28]. As a result, the control and dietary sugar groups exhibited no noticeable difference in LPS (Figure 4f). Phenolic acids and flavonoids in honey, such as chrysin, quercetin, and galangal, may also inhibit the activity of pro-inflammatory enzymes [29]. Reactive oxygen species (ROS) have high oxidation activity and are common byproducts of oxygen metabolism. Increased ROS levels cause significant damage to all biological macromolecules (proteins, lipids, and nucleic acids) [30]. The findings of this experiment are consistent with those of studies conducted with Manuka honey, which has the function of reducing the level of inflammatory cytokines (TNF-α) and increasing antioxidant enzymes (SOD) and non-enzymes (GSH) [31]. Honey, but not sugar, benefits energy metabolism and inhibits inflammation and oxidative stress.

We showed that the abundance of the genus *Muribaculaceae* in the low-dose honey diet was notably higher than that in the low-dose sugar diet (Figure 3d). *Muribaculaceae* is a butyrate-producing bacterium that is downregulated after high-fat diet feeding [11]. The present study showed that an elevated abundance of *Muribaculaceae* causes a reduction in OGTT glucose levels (Figure 5a). The honey and sugar diets induced gut microbiota changes at the genus level, including an increased abundance of *Lachnospiraceae_NK4A136* group (Figure 3c). Furthermore, the *Lachnospiraceae_NK4A136* group in the low-dose sugar diet was notably higher than that in the low-dose honey diet (*p* < 0.05) (Figure 3d). The *Lachnospiraceae_NK4A136* group belongs to the family *Lachnospiraceae*, and the *Lachnospiraceae_NK4A136* group was reported to be decreased in high-fat diet-induced obese mice [32]. In this study, the *Lachnospiraceae_NK4A136* group was found to be positively related to HDL-C (Figure 5a). Short-chain fatty acids can enhance the HDL-C concentration in hypercholesterolemic apolipoprotein E-knockout rats. Moreover, short-chain fatty acids play a significant role in glucose homeostasis, lipogenesis regulation, and energy metabolism. In addition, *Desulfovibrionaceae* in the high-dose sugar diet was markedly higher than in the high-dose honey diet (*p* < 0.05) (Figure 3e), and it was found to be negatively correlated with GSH (Figure 5a). The relative abundance of *Proteobacteria* was higher in patients with metabolic diseases, such as hyperglycemia and obesity. Many members of the Proteobacteria phylum, such as the genus *Desulfovibrionaceae*, can produce LPS as endotoxin-producing pathogens. LPS-binding proteins can trigger the expression and production of proinflammatory cytokines and cause insulin resistance [33]. Figure 3d showed that the genus *Roseburia* in the low-dose honey diet was notably lower than that in the low-dose sugar diet. Studies have shown that *Roseburia* can produce short-chain fatty acids [34]. The abundance of *Roseburia* is reduced in high-fat diet-fed mice and obese humans, and an elevated relative abundance may reduce obesity.

We found that serum oleamide levels were higher in mice fed an HL diet than those fed an FGSL diet (Figure 4c). Oleamide significantly inhibited lipopolysaccharide-induced nitrite production and pro-inflammatory cytokines (TNF-α, IL-6, and IL-1β), thereby attenuating the inflammatory response in RAW264.7 mouse macrophages and reducing paw oedema in a rat model of carrageenan-induced inflammation [35]. A member of the fatty acid amide family, oleamide, is a known modulator of intestinal motility and known for its role in regulating intestinal motility in mice by activating cannabinoid receptor one [36]. It is possible that MaZiRenWan (a Chinese herbal medicine) can improve defecation in mice by enhancing fatty acid amide hydrolase-mediated oleamide degradation [37]. Phospholipids are compounds composed of 1,2-diacylglycerol and a phosphodiester bridge, connecting the glycerol backbone to choline and ethanolamine, which forms phosphatidylcholine (PC) and phosphatidylethanolamine (PE). They can be involved in the composition of cell membranes and mitochondrial membranes in mammals [38]. FGSH-fed mice had higher levels of PE (18:4(6Z,9Z,12Z,15Z)/24:1(15Z)) and lower levels of PC (22:6(4Z,7Z,10Z,13Z,16Z,19Z)/18:0) and PC (22:6(4Z,7Z,10Z,13Z,16Z,19Z)/14:0) than HH-fed mice (Figure 4f). A lower PC: PE ratio was found to be beneficial for reducing liver lipid accumulation in mice and decreasing the harmful effects of inflammation and fibrosis. Concurrently reducing the PC:PE ratio favors glucose metabolism, slows symptoms in genetically obese and insulin-resistant mice, and prevents the development of hepatic insulin resistance. The HL diet resulted in lower levels of 3b,7a,12a-trihydroxy-5b-cholanoic acid (a bile derivative) than the FGSL diet (Figure 4c). Bile acid release may promote the regeneration of intestinal stem cells and epithelial cells and reduce the severe symptoms of inflammatory bowel disease [39].

Finally, we must note that the present experimental design had some limitations. First, we used only male ICR mice and ignored whether there was significant variability in the dietary effects of honey and mixed sugar for female mice. Moreover, we did not monitor the feces of the mice, and therefore, cannot ensure that the honey diet slowed weight gain in relation to nutrient absorption. We will use male and female mice together in subsequent experiments to reduce gender error. Furthermore, the daily fecal discharge of mice will be recorded in a timely manner to better speculate on the metabolic differences in the absorption of honey and mixed sugars in vivo. We expect to reveal the extrinsic and intrinsic associations of honey and mixed-sugar diets in normal mice from a systems biology perspective, which will provide a reference for subsequent clinical studies.

## 5. Conclusions

To test whether replacing sugar with honey in the diet has potential nutritional benefits, we fed mice with natural honey, and compared these mice to those fed an analog sugar diet (mixed fructose, glucose, and sucrose (FSG) solutions) at set dosages for one month. We showed that the honey diet resulted in less weight gain with fewer ballooned hepatocytes. Increased ballooned hepatocytes were found in FGS-fed mice compared to the hepatocytes in mice fed a regular or honey diet. Honey diets decreased serum T-CHO, TGs, and TNF-α and increased HDL-C and antioxidant enzyme levels. We further found that each diet type has distinct gut microbiota and metabolomics profiles. The low-dose honey diet increased *Muribaculaceae* levels in the intestine of mice and reduced serum fatty acid levels. Finally, systems biology approaches were implemented to comprehensively interpret the analysis above. This experiment revealed the extrinsic and intrinsic links between honey and sugar diets in normal mice from the systems biology perspective and provides a reference for follow-up clinical research.

## Figures and Tables

**Table 1:** Animal groups and doses.

Group	Treatment	Dose (g/kg)	Honey Dilution (mg/mL)	Volume (mL)
NC	Control diet	0	0	~0.2
HL	Honey low-dose	1	100	~0.2
HH	Honey high-dose	2	200	~0.2
FGSL	Mixed sugar low-dose	1	100	~0.2
FGSH	Mixed sugar high-dose	2	200	~0.2

## Data Availability

Several data, which were generated during the current study, are not publicly available, but are available from the corresponding author on reasonable request.

## Appendix A

***Single dose:*** 18–20 g male ICR mice were obtained from Beijing Vital River Laboratory Animal Technology Co., Ltd. and housed under controlled temperature (25 °C) and light conditions (12 h light, 12 h dark cycles). After three days of acclimatization, the mice were randomly divided into experimental groups (NC and HL, HH, FGSL, FGSH; n = 8) according to their weight and blood sugar (Table A1). The mice started fasting at 8:00, and the mice in each group were given the corresponding drugs at 14:30. Then, the blood glucose values at 0, 10, 20, 30, 40, 60, and 120 min were measured with an Accu-Chek Active Blood Glucose Meter (Figure A1 and Figure A2).

**Table A1:** Animal groups and dosages of single dose.

Group	Treatment	Dose (g/kg)	Honey Dilution (mg/mL)	Volume (mL)
NC	Control diet	0	0	~0.2
HL	Honey low-dose	2	200	~0.2
HH	Honey high-dose	4	400	~0.2
FGSL	Mixed sugar low-dose	2	200	~0.2
FGSH	Mixed sugar high-dose	4	400	~0.2
